# Improving the Tribological and Anticorrosion Performance of Waterborne Polyurethane Coating by the Synergistic Effect between Modified Graphene Oxide and Polytetrafluoroethylene

**DOI:** 10.3390/nano10010137

**Published:** 2020-01-12

**Authors:** Tao Bai, Lei Lv, Weiping Du, Wenqi Fang, Yansong Wang

**Affiliations:** 1 College of Mechanical Engineering, Donghua University, Shanghai 201620, China; 15269322313@163.com; 2 Engineering Research Center of Advanced Textile Machinery, Ministry of Education, Shanghai 201620, China; 3 College of Materials Science and Engineering, Donghua University, Shanghai 201620, China; duweiping@dhu.edu.cn; 4 Baosteel Research Institute, Baoshan Iron and Steel Co., Ltd., Shanghai 201900, China; wqfang@baosteel.com; 5 Shanghai Weixing Optical Co., Ltd., Shanghai 201404, China

**Keywords:** graphene oxide, waterborne polyurethane, tribological performance, corrosion resistance, electrochemical impedance spectroscopy (EIS)

## Abstract

In this work, the effect of modified graphene oxide and polytetrafluoroethylene (PTFE) on the tribological and anticorrosion properties of waterborne polyurethane (WPU) was studied. The modified graphene oxide (MGO) was obtained by the surface functionalization modification of graphene oxide (GO) with isophorone diisocyanate (IPDI), and MGO/WPU composite coating and MGO-PTFE/WPU composite coating with different mass fractions of MGO were prepared. The tribological and electrochemical experiment results demonstrated that the tribological properties of the coating and the corrosion resistance of the worn coating were effectively enhanced under the synergistic effect of MGO and PTFE. Finally, a mechanism was proposed to explain the improvement in anticorrosion performance of the worn coating.

## 1. Introduction

Friction and corrosion have always been two key factors affecting the life of metal products. Especially after surface damage, metal products are more likely to fail prematurely due to corrosion. To solve this problem, many organic coatings with antifriction and/or anticorrosion properties have emerged. Among various polymers, waterborne polyurethane (WPU) has received much attention due to its low volatile organic compounds (VOC), environment-friendly nature, excellent mechanical properties, and good chemical stability properties [1,2,3,4]. However, the hardness, water resistance, and corrosion resistance of the waterborne polyurethane coating are deteriorated because of the introduction of hydrophilic groups, which greatly limits its development and application. Therefore, it is of great significance for waterborne polyurethane to improve its comprehensive performance by modification.

Graphene, as a two-dimensional nanomaterial, has attracted wide attention due to its unique characteristics such as large specific surface area, excellent mechanical strength, low chemical reactivity, high thermal conductivity, and electrical conductivity [5,6,7]. Recently, many researchers have begun to improve the comprehensive performance of WPU by adding graphene and its derivatives (graphene oxide and functionalized graphene). Wang et al. [8] prepared functionalized graphene-reinforced polyurethane nanocomposite coatings by using the sol-gel method, the experimental results showed that both tensile strength and Young’s modulus of the coating increased. Wan et al. [9] prepared a series of self-healing waterborne polyurethane/graphene oxide nanocomposites by solution blending method, the experimental results showed that the nanocomposites exhibited excellent thermo stability and tensile strength. Zhang et al. [10] modified the GO via self-prepared polymer and fabricated WPU/FGO composites, the experimental results showed that the water resistance and the tensile strength of WPU/FGO films were significantly improved.

Polytetrafluoroethylene (PTFE) also has excellent self-lubricity and has been widely used as a lubricant additive for organic coatings [11]. Moreover, Masood et al. [12] found that graphene nanoplatelets and polytetrafluoroethylene can synergistically improve the tribological properties and adhesion of nylon-based composites. Zhao et al. [13] found that the modified graphene oxide and PTFE can significantly improve the tribological properties of epoxy resins through synergistic effects. Although there are many studies on the effect of the synergistic effect of modified graphene and PTFE on the tribological properties of the coating, little research has been done on the effect of their synergy on the corrosion resistance of worn coatings.

In this study, the modified graphene oxide (MGO) was prepared by the surface functionalization of graphene oxide (GO) with isophorone diisocyanate (IPDI) as modifier [14,15]. MGO/WPU composite coatings and MGO-PTFE/WPU composite coatings with different mass fractions of MGO were prepared by using WPU as matrix polymer, MGO, and nano-scale PTFE as additives. The tribological properties of the coating and the electrochemical behavior of the worn coating were tested. The mechanism of the synergistic effect of MGO and PTFE on tribological performance and corrosion resistance of the coating was studied.

## 2. Experimental Details

### 2.1. Reagents and Materials

Graphene oxide (GO, thickness 4–8 nm) was provided by Suzhou Tanfeng Technology Ltd. (Jiangsu, China). Polytetrafluoroethylene nano powder (PTFE, average particle size 20 nm) was brought from Xincheng Engineering Plastics Ltd. (Guangdong, China). Waterborne polyurethane (WPU) was purchased from Anda Huatai New Material Ltd. (Anhui, China). *N*,*N*-dimethylformamide (DMF, AR, ≥99.0%), Isophorone Diisocyanate (IPDI, AR, 98%), Dichloromethane (DCM, AR, ≥99.5%) and Sodium chloride (NaCl, AR, ≥99.5%) were purchased from Sinopharm Chemical Reagent Ltd. (Shanghai, China)

### 2.2. Preparation of Modified Graphene Oxide

First, 0.2 g GO and 20 mL DMF were added into a flat-bottom three-necked bottle and mixed uniformly, 1.78 g IPDI was added to the above solution, and the obtained mixture was allowed to stir for 24 h in a protective nitrogen gas atmosphere. Then, the product obtained in the previous step was centrifuged, and washed 4 times with DCM to remove unreacted reagent. Finally, MGO could be obtained after desiccation in a vacuum oven for 5 h at 70 °C.

### 2.3. Characterization

The functional groups on the surface of GO and MGO were determined by Fourier transform infrared spectroscopy (FTIR, Nicolet 6700, ThermoFisher Scientific, Waltham, MA, USA) with a scan range of 4000 cm^−1^ to 400 cm^−1^. The molecular structures of GO and MGO are characterized by X-ray diffraction (XRD, D/MAX-2550 PC, Rigaku, Tokyo, JPN) with a ray wavelength of 1.54056 Å and a scan range of 5° to 60°. The defect density of GO and MGO were identified by Raman spectroscopy (Micro-Raman, inVia-Reflex, Renishaw, London, UK) with an excitation wavelength of 523 nm. The content of C, H, and N elements of GO and MGO was analyzed by Elemental analyzer (EA, Vario EL III, Elmentar, Munich, GER). The morphology of wear scars on the surface of the coating and the dispersion state of MGO at the cross section of the coating were observed by the Field emission scanning electron microscope (FESEM, S-4800, Hitachi, Tokyo, JPN).

### 2.4. Preparation of MGO/WPU Composite Coating and MGO-PTFE/WPU Composite Coating

304 stainless steel (304 SS) was selected as the matrix material due to its poor corrosion resistance in environments containing chloride ion, and the oxide film on the 304 SS surface was needed to be removed by sandpaper before coating.

MGO at different mass fractions (0 wt.%, 0.5 wt.%, 1.0 wt.%, 1.5 wt.%, 2.5 wt.%, 3.5 wt.%) was added into 1 g WPU, then those composite solutions were mixed and further dispersed for 6 h by ball milling. The obtained mixture was coated on the surface of 304 SS and cured at room temperature, the MGO/WPU composite coating could be obtained after further heating at 105 °C.

MGO at different mass fractions (0 wt.%, 0.5 wt.%, 1.0 wt.%, 1.5 wt.%, 2.0 wt.%) and PTFE at constant concentration (5.0 wt.%) were added into 1 g WPU, then those composite solutions were mixed and further dispersed for 8 h by ball milling. The obtained mixture was coated on the surface of 304 SS and cured at room temperature, the MGO-PTFE/WPU composite coating could be obtained after further heating at 105 °C.

### 2.5. Tribological Measurements

The tribological performance of MGO/WPU composite coating and MGO-PTFE/WPU composite coating was studied by the high-speed reciprocating friction and wear testing tribometer (HSR-2M, Zhong Ke Kai Hua Corporation, Gansu, China). In tests, GCr15 steel ball with 3 mm radius was selected as a friction pair, the sliding speed was 220 t/min, the reciprocating distance was 5 mm. Due to the different tribological properties of the two composite coating, the applied load and friction time of MGO/WPU composite coating were 3.5 N and 5 min respectively, and the applied load and friction time of MGO-PTFE/WPU composite coating were 6.0 N and 20 min respectively. In addition, five independent repeated experiments were performed for each composite coating with same mass fraction.

### 2.6. Electrochemical Measurements

Electrochemical impedance spectroscopy (EIS) is a commonly method for testing the corrosion resistance of organic coatings. The corrosion resistance of MGO/WPU composite coating and MGO-PTFE/WPU composite coating after rubbing was studied by electrochemical analyzer (CHI600D, Shanghai Chenhua Instrument Ltd., Shanghai, China) at room temperature. The test solution was NaCl solution with a mass fraction of 3.5 wt.%, the working electrode was the 304 SS with worn composite coating, the counter electrode was platinum wire and the reference electrode was saturated calomel electrode (SCE).

First, the open circuit potential (OCP) was needed to be monitored continuously to obtain a steady potential. Then, the EIS test was performed under the condition that the frequency range was 10^−2^ Hz–10^5^ Hz, the alternating voltage was 10 mv, and other parameters were default values. Finally, the ZSimpWin 3.60 software (Bruno Yeum, Ph.D., Ann Arbor, Michigan, MI, USA) was used for data analysis to determine equivalent circuit.

## 3. Results and Discussion

### 3.1. FTIR Characterization

**Figure 1:**
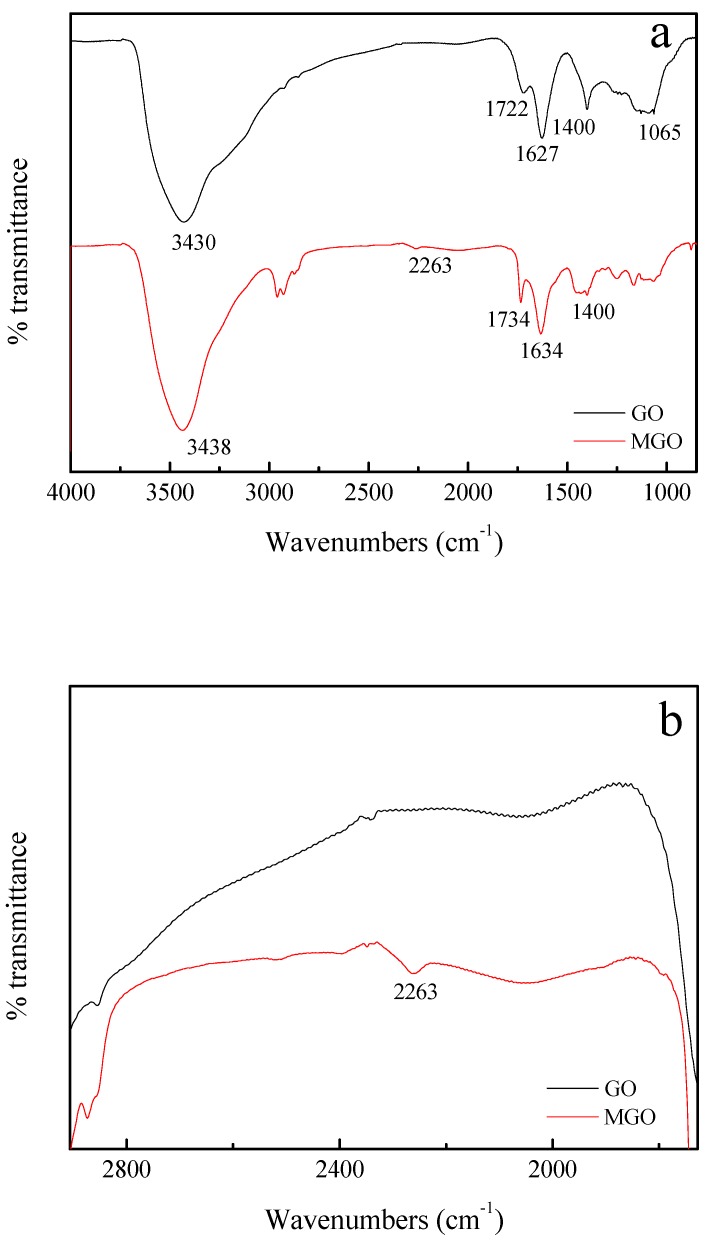
FTIR spectra (**a**) and partially enlarged views (**b**) of GO and MGO.

The FTIR spectra of GO and MGO are shown in Figure 1. In Figure 1a, the absorption peaks of GO at 3430 cm^−1^, 1722 cm^−1^, 1627 cm^−1^ and 1065 cm^−1^ respectively represent the stretching vibration of O-H, the stretching vibration of C=O, the skeleton vibration of C=C and the stretching vibration of C-O, and the absorption peaks of GO at 1400 cm^-1^ represent the bending vibration of C-OH. After modification, it can be observed clearly in Figure 1b that a new absorption peak at 2263 cm^−1^ represents the isocyanate group (-NCO) [14,15], and a triple peak at ~3000 cm^−1^ represent the symmetric and asymmetric stretching vibrations of -CH_2_ and -CH_3_ groups [16], which indicates that GO was successfully modified by IPDI. Moreover, the absorption peaks of C=C skeleton vibration at 1634 cm^−1^ appears indicating that the basic structures of MGO have not changed.

### 3.2. XRD Characterization

**Figure 2:**
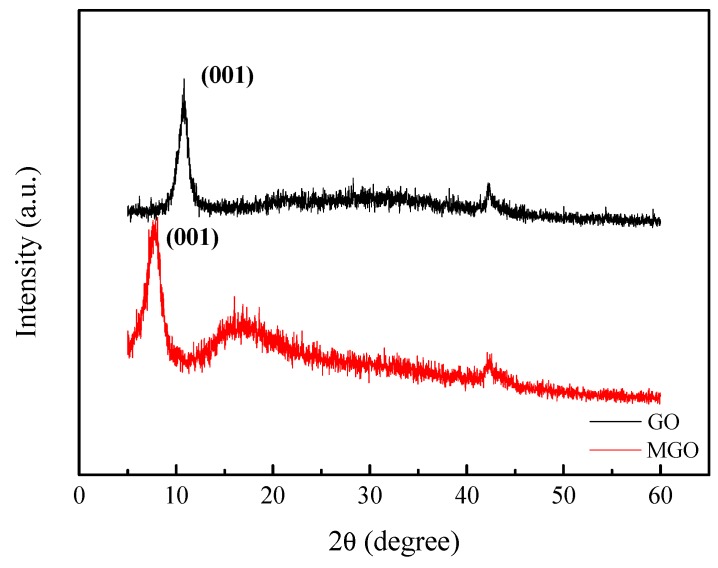
XRD spectra of GO and MGO.

The XRD was performed to study the molecular structure of GO and MGO as shown in Figure 2. It can be observed from Figure 2 that GO and MGO have a strong diffraction peak, and the peak represents the diffraction peak on the (001) crystal plane. After the modification of GO with IPDI, the diffraction peak shifts from *2θ* = 10.9° (*d* = 8.1Å) to the left to *2θ* = 7.8° (*d* = 11.3Å), the enlargement of interlayer space of MGO is due to the IPDI linkage to the surface of GO. Moreover, the broad diffraction peak around *2θ* = 13–25° indicates a smaller interlayer spacing, suggesting the removal of oxygen-rich groups on the GO surface [17].

### 3.3. Raman Characterization

**Figure 3:**
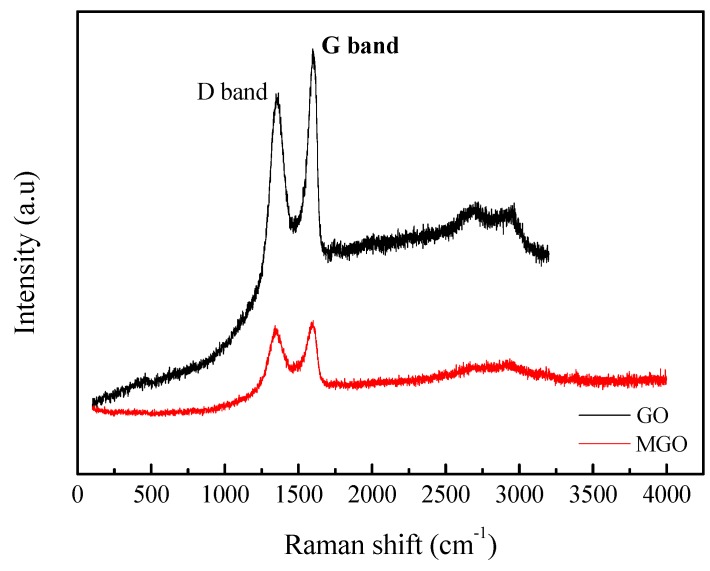
Raman spectra of GO and MGO.

The Raman spectra of GO and MGO are shown in Figure 3. In Figure 3, there are two peaks near ~1350 cm^−1^ and ~1600 cm^−1^ representing D and G peaks, respectively. The defect density can be obtained by calculating the intensity ratio of the D peak to the G peak (*I_D_*/*I_G_*), and the larger the *I_D_*/*I_G_* value, the greater the defect density [18,19]. The results show that the *I_D_*/*I_G_* ratio of GO is 0.92, the *I_D_*/*I_G_* ratio of MGO is 0.96, the increase in the defect density also indicates the successful covalent bonding of IPDI and GO.

### 3.4. Elemental Analysis

The contents of C, H, and N in GO and MGO are shown in Table 1. It can be seen that the content of N in GO is less than 0.05%, while the content of N in MGO increases to 9.87%, which indicates that there are many isocyanate groups (-NCO) on the surface of MGO. This result is consistent with the analytical results of FTIR spectra, further demonstrating that the modification is successful.

### 3.5. Tribological Performance of the MGO/WPU Composite Coating

**Figure 4:**
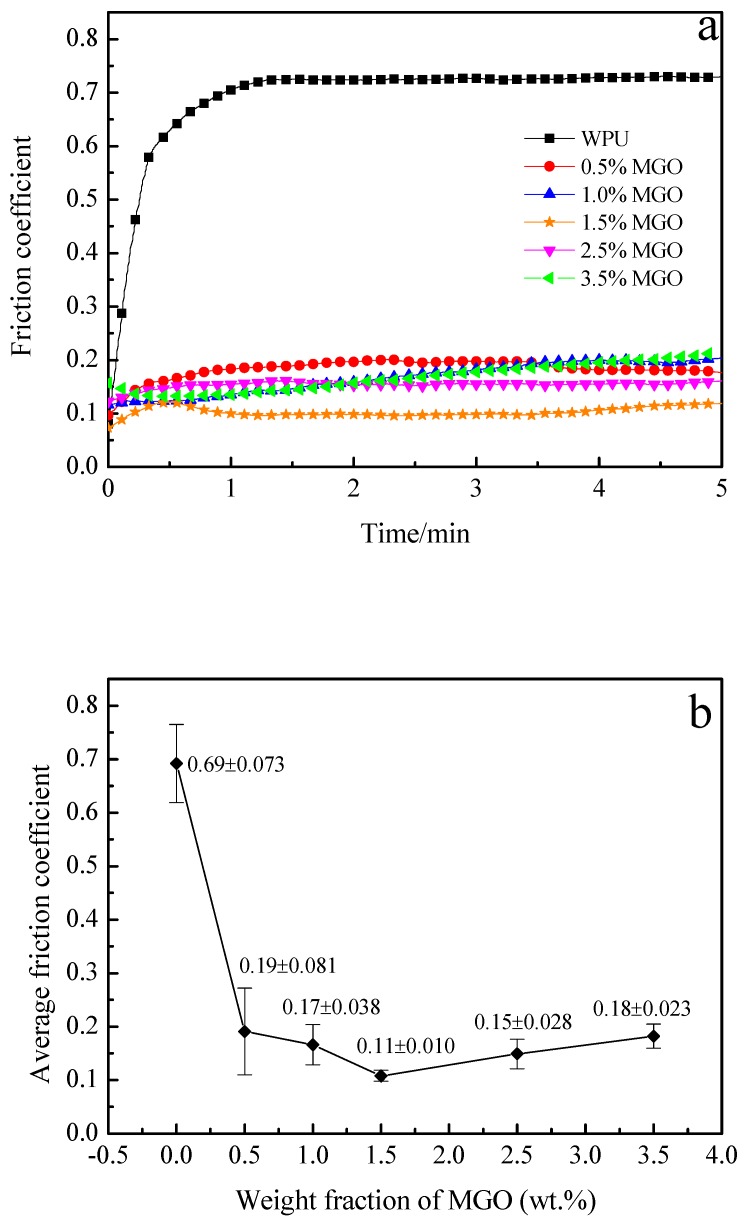
Friction coefficient of MGO/WPU composite coating with different mass fractions of MGO, (**a**) real-time friction coefficient, (**b**) average friction coefficient. (Applied load is 3.5 N; test duration is 5 min)

To study the effect of MGO on the tribological performance of WPU coating, the friction coefficient of MGO/WPU composite coating with different mass fractions of MGO is tested, the results are shown in Figure 4.

It can be found from Figure 4 that friction coefficient of pure WPU coating increases sharply at the beginning of the experiment, and then gradually stabilizes at around 0.69. When MGO is added into pure WPU, the friction coefficient of MGO/WPU composite coating reduces, with the increase of MGO mass fraction, the friction coefficient of coating decreases first and then increases. When the mass fraction of MGO is 1.5 wt.%, the friction coefficient of the composite coating reaches a minimum of 0.11. The -NCO functional group grafted on the surface of MGO can weaken the van der Waals force between its particles, which promotes the uniform dispersion of MGO in the WPU, so that MGO can form an effective transfer film on the surface of friction pair during the experiment. Moreover, the force between the MGO sheets is small and the relative sliding is liable to occur, so that the composite coating exhibits excellent antifriction performance.

**Figure 5:**
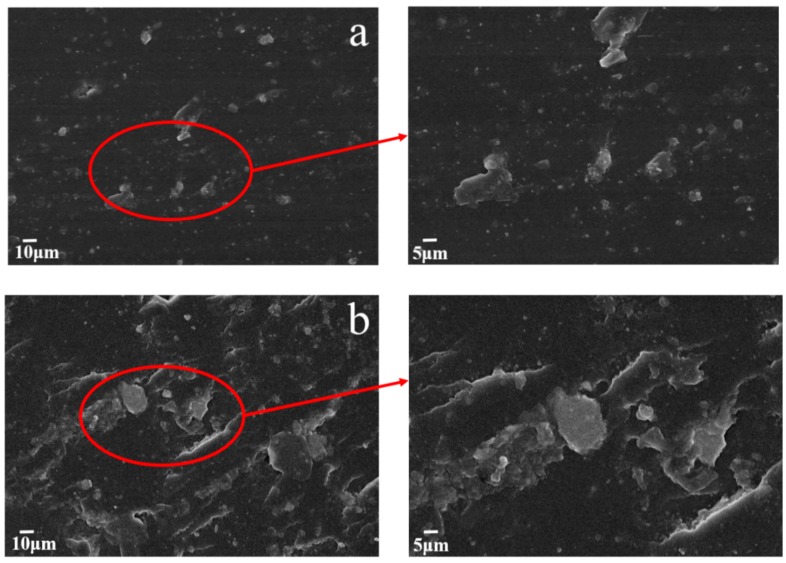
Micrographs of the dispersion state of MGO on the cross section of the MGO/WPU composite coating with different contents of MGO, (**a**) 1.5 wt.%, (**b**) 3.5 wt.%.

However, when the mass fraction of MGO exceeds 1.5 wt.%, the friction coefficient of the MGO/WPU composite coating begin to increase. The higher the concentration of MGO, the more likely it is to agglomerate, and the agglomerated MGO is easily carried away by the friction pair during the friction process, so that a continuous transfer film cannot be formed on the surface of the friction pair [12]. Figure 5 shows micrographs of the cross-sectional of the MGO/WPU coatings with MGO mass fraction of 1.5 wt.% and 3.5 wt.%. As shown in Figure 5b, when the mass fraction of MGO is 3.5 wt.%, MGO severely agglomerates in the coating. Although the Van der Waals forces between the MGO particles is reduced after the modification of IPDI, when MGO is at a high concentration, it is still prone to form agglomeration due to its unstable state of energy state.

**Figure 6:**
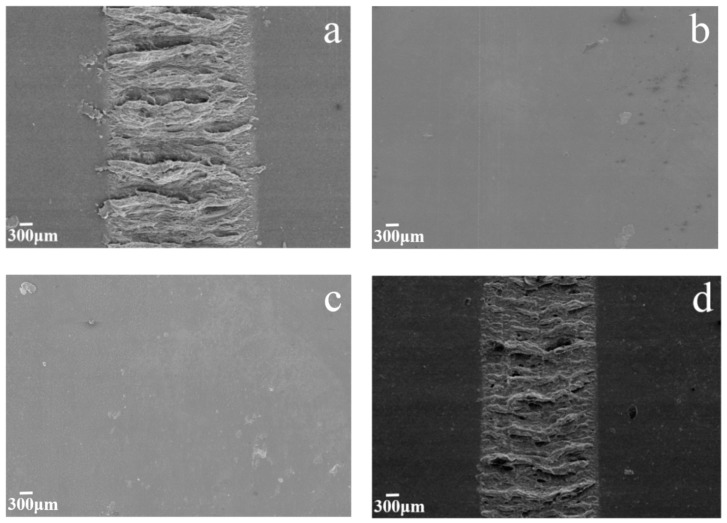
Micrographs of worn surface of MGO/WPU composite coating with different mass fractions of MGO, (**a**) 0.5 wt.%, (**b**) 1.0 wt.%, (**c**) 1.5 wt.%, (**d**) 3.5 wt.%.

The micrographs of worn surface of MGO/WPU composite coating with different mass fractions of MGO after reciprocating friction test are shown in Figure 6. As can be seen from Figure 6a, when the content of MGO is low, the worn surface of composite coating is rough and has severe delamination. As the MGO mass fraction increases, the delamination disappears, and the worn surface of the composite coating gradually becomes smooth, especially when the mass fraction of MGO is 1.5 wt.%, the worn surface of the composite coating is very smooth and has no delamination as shown in Figure 6c. However, when the content of MGO is too high, the worn surface of the composite coating becomes rough again, and the delamination occurs again, as shown in Figure 6d. The -NCO functional group grafted on the surface of the MGO can combine with the carbamate functional group in the WPU to increase the binding force between the MGO and the WPU, thereby adhesion transfer of the matrix resin is effectively reduced during the rubbing process. In addition, uniformly dispersed MGO can form an effective transfer film on the surface of the friction pair, which not only reduces the friction coefficient of the coating, but also decreases the wear of the coating surface.

When the content of MGO is too high, the MGO in the WPU starts to agglomerate, which affects the formation of an effective transfer film on the surface of friction pair. Moreover, the agglomerated MGO can weaken the bonding force between the MGO and the WPU, thereby the surface quality of the coating becomes poor again.

### 3.6. Tribological Performance of the MGO-PTFE/WPU Composite Coating

**Figure 7:**
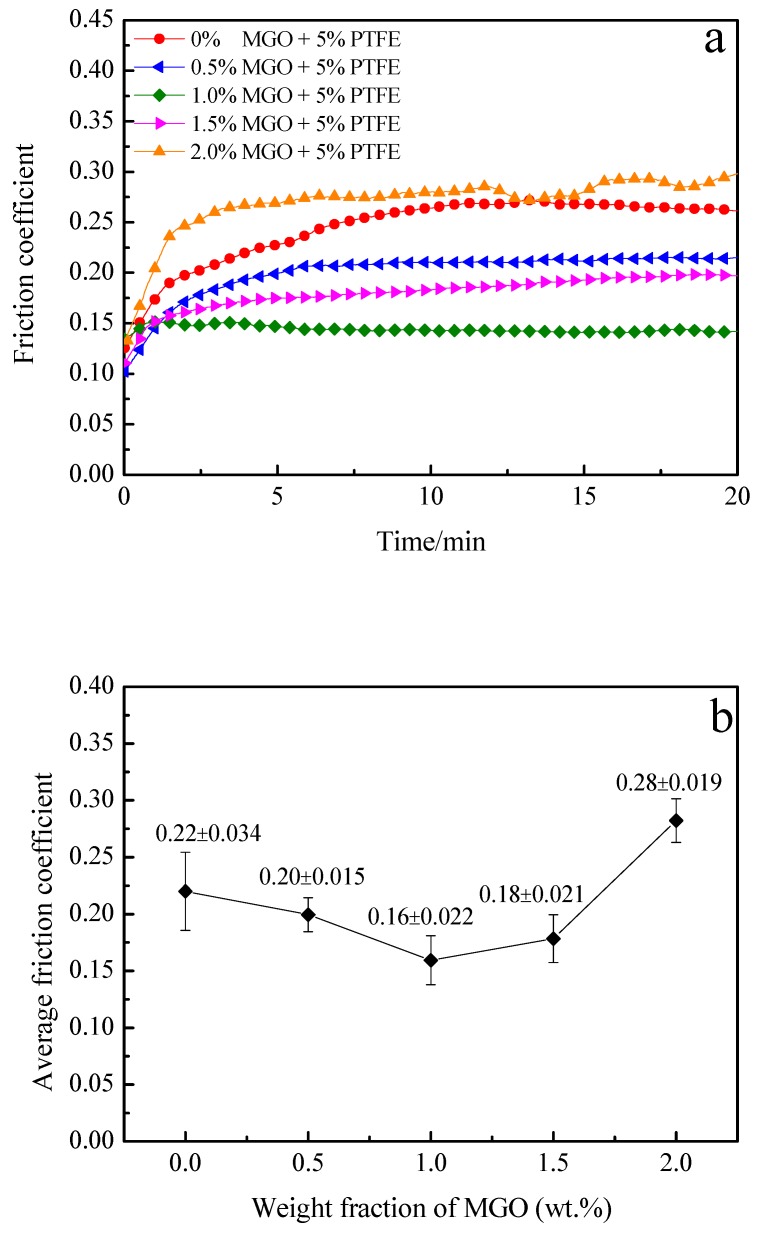
Friction coefficient of MGO-PTFE/WPU composite coating with different mass fractions of MGO, (**a**) real-time friction coefficient, (**b**) average friction coefficient. (Applied load is 6 N; test duration is 20 min)

To explore the influence of synergistic effect of MGO and PTFE on the tribological performance of the coating, the friction coefficient of MGO-PTFE/WPU composite coatings with different mass fractions of MGO are tested, and the results are shown in Figure 7.

It can be observed from Figure 7 that when the mass fraction of MGO is 0 wt.%, the friction coefficient of the composite coating is significantly lower than that of the pure WPU coating, this is because PTFE has excellent self-lubricating properties. With the increase of MGO content, the friction coefficient of MGO-PTFE/WPU composite coating also first decreases and then increases, and when the mass fraction of MGO reaches 1.0 wt.%, the coefficient of friction reaches a minimum value of 0.16. In tribological experiments, the applied load and the test duration of the MGO-PTFE/WPU composite coatings were 6 N and 20 min, but the applied load and the test duration of the MGO/WPU composite coatings were 3.5 N and 5 min. The larger the applied load and the longer the test duration, the better the wear resistance of the coating. Although the average friction coefficient of the MGO-PTFE/WPU composite coating is not significantly reduced compared to that of MGO/WPU composite coating, the wear resistance of the MGO-PTFE/WPU composite coating is greatly improved. Both MGO and PTFE have excellent self-lubricating properties, and PTFE can improve the ability of the MGO-PTFE/WPU composite coating to withstand the applied load. Therefore, under the synergistic effect of MGO and PTFE, even if the applied load is increased and the friction time is prolonged, the MGO-PTFE/WPU composite coating still shows excellent tribological performance.

**Figure 8:**
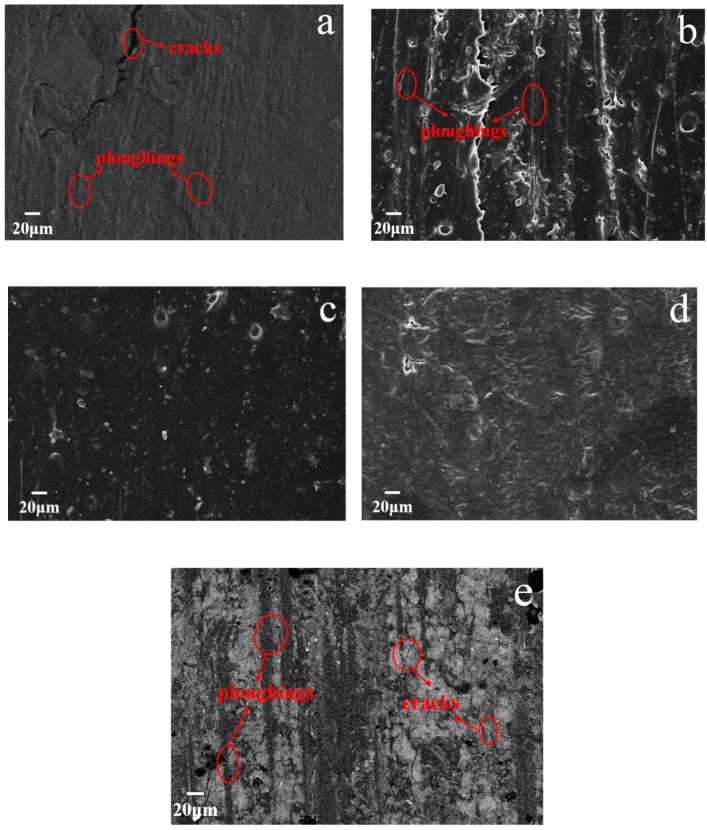
Micrographs of worn surface of MGO-PTFE/WPU composite coating with different mass fractions of MGO, (**a**) 0 wt.%, (**b**) 0.5 wt.%, (**c**) 1.0 wt.%, (**d**) 1.5 wt.%, (**e**) 2.0 wt.%.

Figure 8 presents micrographs of worn surface of MGO-PTFE/WPU composite coating with different mass fractions of MGO after reciprocating friction test. As shown in Figure 8a, there are deep ploughings and cracks on worn surface of the composite coating. When the WPU only contains PTFE, since the bonding force between PTFE and WPU is very weak, micro-cracks are prone to occur at the interface junction between PTFE and WPU and continue to extend under the action of reciprocating pressure, when these micro-cracks connect to the surface, cracks are generated. Moreover, PTFE is easy to agglomerate in WPU, resulting in ploughings on the surface of the coating. As the content of MGO increases, it can be seen from Figure 8b that the cracks on worn surface of the composite coating disappear and ploughings become shallow, especially when the mass fraction of MGO reaches 1.0 wt.%, the worn surface of the composite coating is smooth and has no cracks and ploughings even after friction test of 20 min, as shown in Figure 8c. This indicates that the addition of MGO can enhance the tribological performance of the MGO-PTFE/WPU composite coating. However, when the mass fraction of MGO is 2.0 wt.%, both MGO and PTFE have agglomerated, which will not only weaken the ability of MGO and PTFE to hinder the propagation of micro-cracks, but also reduce the tribological performance of MGO-PTFE/WPU composite coating. Therefore, cracks and ploughings reappear on the surface of the coating as shown in Figure 8e.

### 3.7. Anticorrosion Performance of MGO/WPU Composite Coating and MGO-PTFE/WPU Composite Coating

**Figure 9:**
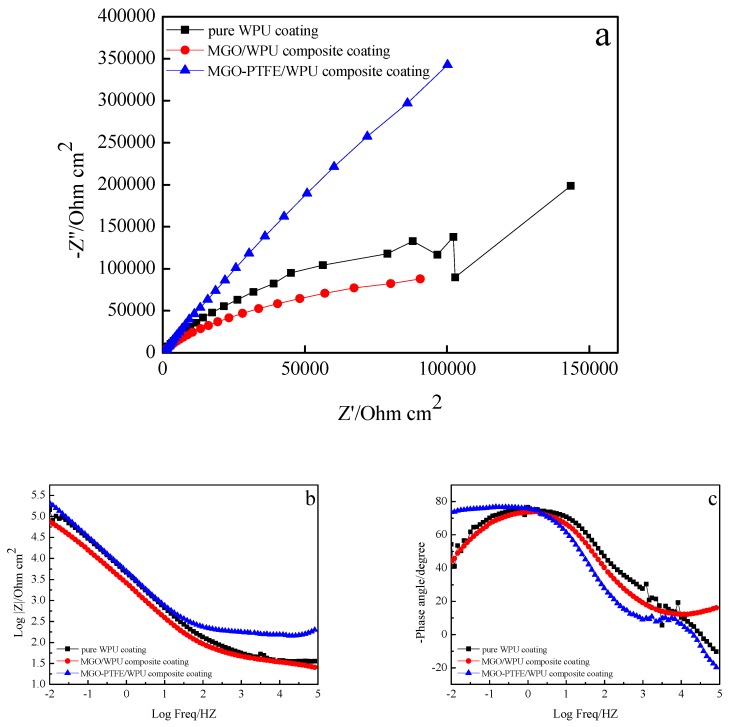
EIS diagrams of pure WPU coating, 0.5 wt.% MGO/WPU composite coating and 0.5 wt.% MGO-PTFE/WPU composite coating after friction, (**a**) Nyquist plot, (**b**) and (**c**) Bode plot.

**Figure 10:**
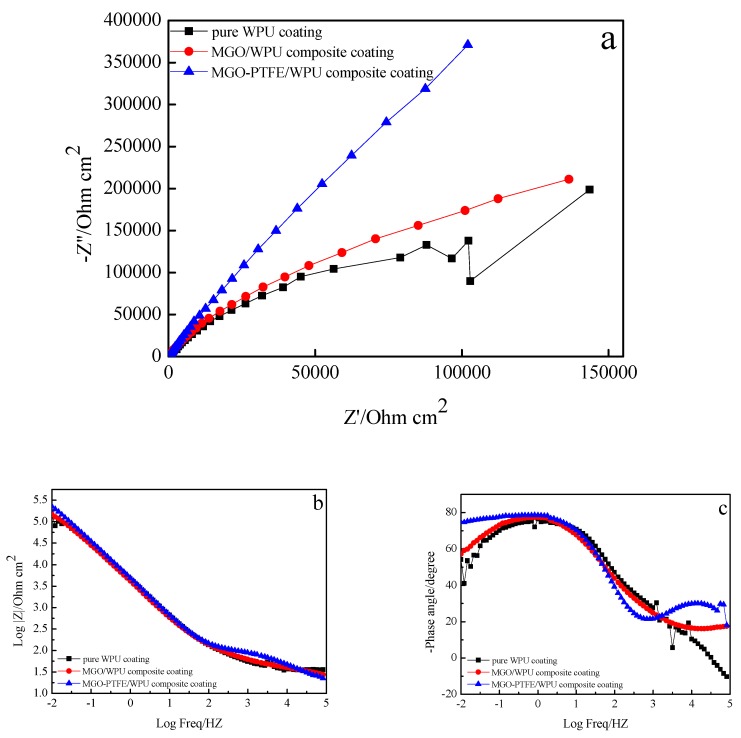
EIS diagrams of pure WPU coating, 1.0 wt.% MGO/WPU composite coating and 1.0 wt.% MGO-PTFE/WPU composite coating after friction, (**a**) Nyquist plot, (**b**) and (**c**) Bode plot.

**Figure 11:**
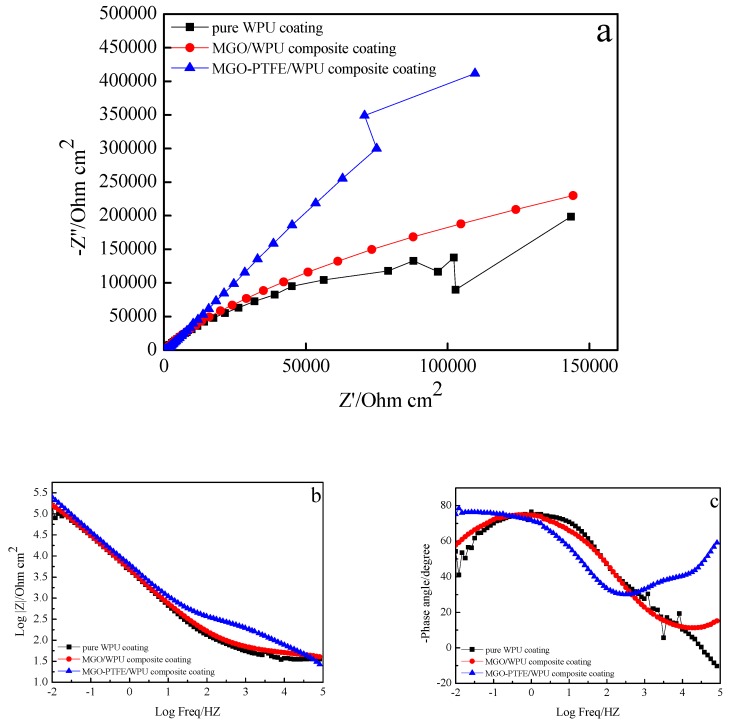
EIS diagrams of pure WPU coating, 1.5 wt.% MGO/WPU composite coating and 1.5 wt.% MGO-PTFE/WPU composite coating after friction, (**a**) Nyquist plot, (**b**) and (**c**) Bode plot.

To study the effects of MGO, MGO, and PTFE on the corrosion resistance of the worn composite coating, the corrosion resistance of pure WPU coating, MGO/WPU composite coating and MGO-PTFE/WPU composite coating after friction was tested by EIS. In this study, three sets of experiments were performed. After the friction test, the corrosion resistance of MGO/WPU composite coatings and MGO-PTFE/WPU composite coatings with different mass fractions was tested respectively, where the mass fraction of MGO was 0.5 wt.%, 1.0 wt.% and 1.5 wt.%, then the corrosion resistance of MGO/WPU composite coatings and MGO-PTFE/WPU composite coatings with the same mass fraction of MGO was compared with that of pure WPU coatings respectively, and the results are shown in Figure 9, Figure 10 and Figure 11 respectively.

**Figure 12:**
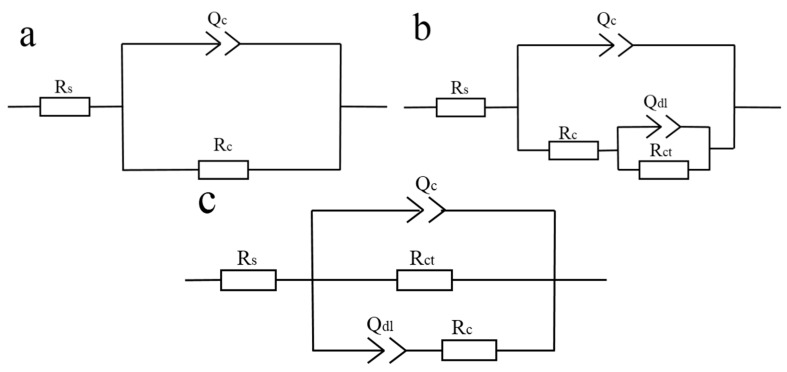
Equivalent circuit for (**a**) WPU coating, (**b**) MGO/WPU composite coating and (**c**) MGO-PTFE/WPU composite coating.

The Nyquist plot is composed by a capacitive arc, the radius of capacitive arc can reflect the rate of the corrosion. The larger the radius of the capacitive arc, the slower the corrosion rate, indicating better corrosion resistance of the composite coating [20]. It can be observed from Figure 9a, Figure 10a and Figure 11a that the radius of the capacitive arc of the MGO-PTFE/WPU composite coating is the largest, indicating that composite coating has the excellent corrosion resistance. Moreover, the impedance modulus (|Z|) at the lowest frequency (0.01 Hz) in Bode plot is usually used as an indicator of coating’s barrier performance, and the larger the impedance modulus, the better the protection performance of the composite coating [21,22]. In Figure 9b, Figure 10b and Figure 11b, it can be observed that the impedance modulus (|Z|) of the MGO-PTFE/WPU composite coating is the largest, indicating that composite coating has the best protection performance. In summary, the MGO-PTFE/WPU composite coating after rubbing shows the best anticorrosion performance. In addition, one time constant can be observed for pure WPU coating, two time constant can be observed for MGO/WPU composite coating and MGO-PTFE/WPU composite coating as shown in Figure 9c, Figure 10c and Figure 11c. To further analyze the electrochemical behavior of the composite coating, the equivalent circuits of the pure WPU coating, the MGO/WPU composite coating and the MGO-PTFE/WPU composite coating were obtained by ZSimpWin software, as shown in Figure 12.

In the proposed equivalent circuit, *R_s_* represents the solution resistance, *R_c_* and *Q_c_* represent the resistance and capacitance of the composite coating, *R_ct_* represents the charge transfer resistance, and *Q_dl_* represents the double layer capacitance. Since the electrode surface is usually non-ideal capacitive response, the constant phase element (*Q*) is introduced [23,24]. The values of *R_c_* and *R_ct_* are two important parameters for evaluating the corrosion resistance of the composite coatings, the larger the *R_c_* and *R_ct_* values, and the better corrosion resistance of the composite coating [25]. Table 2, Table 3 and Table 4 show the fitting data of pure WPU coating and MGO/WPU composite coating and MGO-PTFE/WPU composite coating.

In Table 2, Table 3 and Table 4, it can be found that the *R_c_* and *R_ct_* values of the MGO-PTFE/WPU composite coating are the biggest, and its *R_c_* value is many order of magnitude higher than that of MGO/WPU composite coating and pure WPU coating. The maximum *R_c_* and *R_ct_* values indicate that the MGO-PTFE/WPU composite coating after friction has the best corrosion resistance. Moreover, the *R_c_* value of the pure WPU coating is the smallest, after the addition of MGO, the *R_c_* value of the MGO/WPU composite coating begins to increase. However, when the mass fraction of MGO is 0.5 wt.%, it is found that the *R_c_* value of MGO/WPU composite coating is lower than that of pure WPU coating, this may be because the surface of the composite coating is worn serious after friction, and the content of MGO is low, which causes a lot of corrosive medium penetrate into the coating.

**Figure 13:**
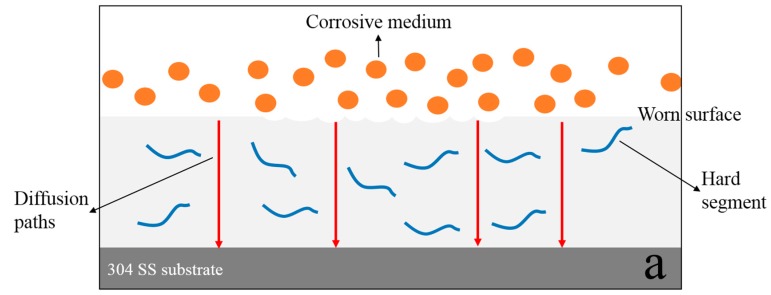

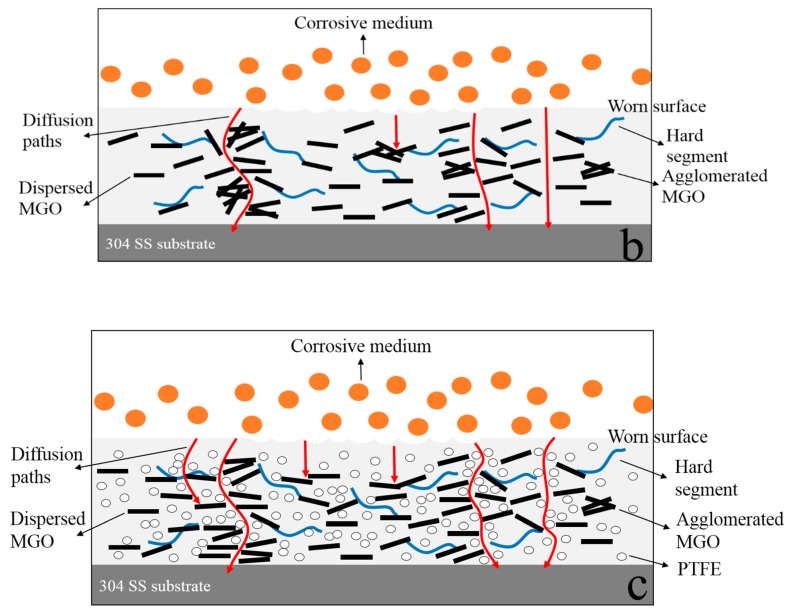
The anticorrosion mechanism for the worn pure WPU coating (**a**), the worn MGO/WPU composite coating (**b**) and the worn MGO-PTFE/WPU composite coating (**c**).

The anticorrosion mechanism for the worn pure WPU coating, the worn MGO/WPU composite coating and the worn MGO-PTFE/WPU composite coating after friction is drawn to intuitively explain the results of electrochemical experiments, as shown in Figure 13. As can be seen from Figure 13a, when no filler is added to the WPU, the corrosive medium directly penetrates the worn coating to corrode the matrix metal. As shown in Figure 13b, MGO left in the MGO/WPU composite coating can act as an excellent barrier to the corrosive medium, and they can zigzag and block the diffusion path of corrosive medium through coating to the metal/coating interface [25,26]. Therefore, the *R_c_* value of the MGO/WPU composite coating increases, and its corrosion resistance is enhanced. After adding MGO and PTFE to the WPU, it can be seen from Figure 13c that the dispersion of MGO left in the WPU is more uniform, and the barrier effect of MGO is further enhanced, blocking many diffusion paths of the corrosive medium. In addition, PTFE can fill the gaps between MGO, which can further increase the tortuosity of the diffusion paths of corrosive medium. Therefore, the MGO-PTFE/WPU composite coating shows the best corrosion resistance, which can be confirmed by its maximum values of *R_c_* and *R_ct_.*

## 4. Conclusions

In this study, MGO was first obtained by surface modification of GO with IPDI. Then MGO/WPU composite coatings and MGO-PTFE/WPU composite coatings containing different mass fractions of MGO were prepared, and their tribological properties and corrosion resistance were tested. Finally, the effect of synergy of MGO and PTFE on tribological properties and anticorrosion performance of the composite coating was studied. The results are as follows:

(1) After the surface modification of GO by IPDI, the -NCO functional group is successfully grafted on the surface of MGO.
(2) The tribological performance of MGO-PTFE/WPU composite coating are significantly improved after the addition of MGO and PTFE. This is because both MGO and PTFE have excellent self-lubricating properties, and PTFE can improve the ability of the MGO-PTFE/WPU composite coating to withstand the applied load. Therefore, under the synergistic effect of MGO and PTFE, the MGO-PTFE/WPU composite coating shows the excellent tribological performance.
(3) MGO left in the worn composite coating can act as an excellent barrier to the corrosive medium, and they can zigzag and block the diffusion path of corrosive medium through coating to the metal/coating interface. After continuing to add PTFE, PTFE can promote the dispersion of MGO, and it can fill the gaps between MGO, which further increases the tortuosity of the diffusion paths of corrosive medium. Therefore, the worn MGO-PTFE/WPU composite coating shows the best corrosion resistance.

## Figures and Tables

**Table 1:** Contents of C, H, and N in GO and MGO.

Sample	C/%	H/%	N/%
GO	45.82	3.07	≤0.05
MGO	58.18	7.73	9.87

**Table 2:** Fitting data of pure WPU coating, 0.5 wt.% MGO/WPU composite coating and 0.5 wt.% MGO-PTFE/WPU composite coating.

	WPU	MGO/WPU	MGO-PTFE/WPU
*R_s_*/(Ω cm^−2^)	6.90 × 10^+1^	4.00 × 10^+1^	2.25 × 10^+1^
*Q_c_-Y_o_*/(Ω^−1^ cm^−2^ s^−n^)	2.93 × 10^−5^	4.20 × 10^−5^	6.02 × 10^−6^
*Q_c_-n*	0.7964	0.8239	0.6969
*R_c_*/(Ω cm^−2^)	7.28 × 10^+5^	2.48 × 10^+5^	3.46 × 10^+13^
*Q_dl_-Y_o_*/(Ω^−1^ cm^−2^ s^−n^)	/	1.02 × 10^−5^	2.65 × 10^−5^
*Q_dl_-n*	/	0.7272	0.8267
*R_ct_*/(Ω cm^−2^)	/	5.06 × 10^+1^	6.01 × 10^+1^

**Table 3:** Fitting data of pure WPU coating, 1.0 wt.% MGO/WPU composite coating and 1.0 wt.% MGO-PTFE/WPU composite coating.

	WPU	MGO/WPU	MGO-PTFE/WPU
*R_s_*/(Ω cm^−2^)	6.90 × 10^+1^	4.79 × 10^+1^	2.75 × 10^+1^
*Q_c_-Y_o_*/(Ω^−1^ cm^−2^ s^−n^)	2.93 × 10^−5^	2.28 × 10^−5^	5.80 × 10^−6^
*Q_c_-n*	0.7964	0.8492	0.7016
*R_c_*/(Ω cm^−2^)	7.28 × 10^+5^	8.61 × 10^+5^	7.37 × 10^+13^
*Q_dl_-Y_o_*/(Ω^−1^ cm^−2^ s^−n^)	/	8.29 × 10^−6^	1.97 × 10^−5^
*Q_dl_-n*	/	0.7583	0.8904
*R_ct_*/(Ω cm^−2^)	/	1.00 × 10^+2^	1.64 × 10^+2^

**Table 4:** Fitting data of pure WPU coating, 1.5 wt.% MGO/WPU composite coating and 1.5 wt.% MGO-PTFE/WPU composite coating.

	WPU	MGO/WPU	MGO-PTFE/WPU
*R_s_*/(Ω cm^−2^)	6.90 × 10^+1^	6.75 × 10^+1^	1.11 × 10^+1^
*Q_c_-Y_o_*/(Ω^−1^ cm^−2^ s^−n^)	2.93 × 10^−5^	2.20 × 10^−5^	4.81 × 10^−6^
*Q_c_-n*	0.7964	0.8051	0.6552
*R_c_*/(Ω cm^−2^)	7.28 × 10^+5^	1.20 × 10^+6^	2.62 × 10^+15^
*Q_dl_-Y_o_*/(Ω^−1^ cm^−2^ s^−n^)	/	5.90 × 10^−6^	1.71 × 10^−5^
*Q_dl_-n*	/	0.7771	0.8238
*R_ct_*/(Ω cm^−2^)	/	1.21 × 10^+2^	5.67 × 10^+2^
